# Influenza transmissibility among patients and health-care professionals in a geriatric short-stay unit using individual contact data

**DOI:** 10.1038/s41598-023-36908-5

**Published:** 2023-06-29

**Authors:** Marie-Paule Gustin, Laurent Pujo-Menjouet, Philippe Vanhems

**Affiliations:** 1 Department of Public Health, Institute of Pharmacy, CIRI-Centre International de Recherche en Infectiologie, Inserm, U1111, CNRS, UMR 5308, ENS Lyon, Equipe PHIE3D, University Lyon, University Claude Bernard Lyon 1, 7 Rue Guillaume Paradin, 69372 Lyon, France; 2 University of Lyon, University Claude Bernard Lyon 1, CNRS UMR5208, Inria, Dracula Team, Institut Camille Jordan, 69622 Villeurbanne, France; 3 Hospices Civils de Lyon, Service Hygiène, CIRI-Centre International de Recherche en Infectiologie, Université Lyon, Université Claude Bernard Lyon 1, Inserm, U1111, CNRS, UMR5308, ENS Lyon, Lyon, France

**Keywords:** Epidemiology, Dynamical systems, Public health, Infectious diseases

## Abstract

Detailed information are lacking on influenza transmissibility in hospital although clusters are regularly reported. In this pilot study, our goal was to estimate the transmission rate of H3N2 2012-influenza, among patients and health care professionals in a short-term Acute Care for the Elderly Unit by using a stochastic approach and a simple susceptible-exposed-infectious-removed model. Transmission parameters were derived from documented individual contact data collected by Radio Frequency IDentification technology at the epidemic peak. From our model, nurses appeared to transmit infection to a patient more frequently with a transmission rate of 1.04 per day on average compared to 0.38 from medical doctors. This transmission rate was 0.34 between nurses. These results, even obtained in this specific context, might give a relevant insight of the influenza dynamics in hospitals and will help to improve and to target control measures for preventing nosocomial transmission of influenza. The investigation of nosocomial transmission of SARS-COV-2 might gain from similar approaches.

## Introduction

Seasonal influenza can cause critical illnesses, hospitalisation and even death, especially in the elderly and people with severe underlying diseases. Patients falling into these categories were shown to have about a 1.5 times higher risk of death related to influenza-like illness (ILI)^1^. According to the Canadian Nosocomial Infection Surveillance Program, the influenza A (H1N1) caused a sharp rise in the 30-day mortality attributable to influenza starting from 25 to 62% after the pandemic of 2009^2^. The impact on medical costs is substantial while hospitalisation of influenza-confirmed patients lasted significantly longer compared with other patients (12.3 days vs. 24.7 h)^3^. During seasonal influenza, a large number of admitted influenza-confirmed patients with a subsequent long length of stay provide a favourable context for nosocomial transmission. This consequently needs appropriate control measures^4,5^.

During flu season, nosocomial outbreaks of influenza are reported in different patient populations (i.e. in Intensive Care Units, in internal medicine wards, in surgery wards, in geriatric units) but also within health care professionals (HCP)^5^. Hospitalized influenza-infected patients lead to a significant amount of extra work for HCP for controlling potential viral spread and disorganisation of care might occur. Then, the contact patterns for care will influence the risk of transmission between patients and HCP but also within each population (patient, nurse or medical doctor).

Models of transmission of healthcare associated infections will help to guide hospital infection control policies and to simulate the prevention effects^6^. Van Der Dool et al. used a stochastic agent-based model and contacts between HCP or patients in order to study i) the effect of vaccination of HCP on influenza infection among patients^7^ and ii) the effect of oseltamivir prophylaxis without and with resistant influenza virus strain^8^. With the same objectives, dynamic modelling, applied to COVID-19, was recently used in a hospital setting. Smith et al. analysed surveillance strategies against COVID-19 using agent-based model and electronically collected contact data from the I-Bird project in long-term care facility^9,10^.

**Figure 1:**
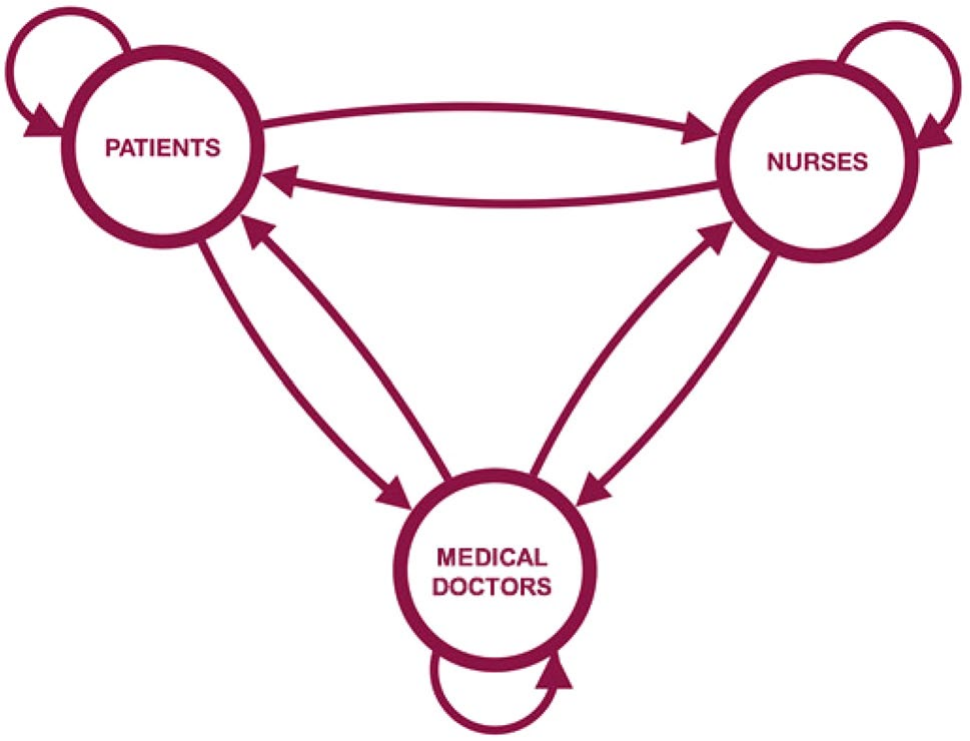
Different types of contacts from a contagious individual towards a susceptible individual.

In a hospital environment, no one is expected to be equally in contact with every other individual: the contact rate between subjects will depend on their roles. Patients and HCP mixed differently because of their status and related daily tasks. Therefore, the potential transmissibility of influenza between patients and different categories of hospital staff has to be precisely quantified to suggest the best strategies to reduce the viral spread. Figure 1 illustrates the 9 possible ways an infection can be transmitted from a contagious individual towards a susceptible one during contact according to their roles: patients, nurses and medical doctors. These 9 transmission patterns could be estimated and written in a 3 × 3 "who acquires infection from whom" matrix of transmission rates ($\beta$ matrix). It should be noted that transmission rates ($\beta$s) are derived from the number of contacts and the probability that the transmission of infection occurs when a susceptible and a contagious individual enter into contact (probability of transmission given contact). This probability varies according to the duration of the contact^11^.

The numbers of contact in a heterogeneous population are commonly estimated either from self-report logs, questionnaires, surveys^7,8^ or contacts recorded by proximity-sensing technology based on wearable sensors^10,12,13^. Some studies comparing methods have highlighted the most objective and exhaustive information provided by wireless sensors^14–16^. It has been shown that only 72% of short contacts (< 5 min) recorded by wireless sensors were actually reported by the participants^16^. In infectious disease modelling, the probability of transmission given contact is usually assumed^17^, drawn from the literature^9^ or fitted so that it is possible, from the model, to estimate a basic reproduction number ${R}_{0}$ compatible with those observed in the literature^7^.

The objective of this pilot study was to develop a dynamical model that allows us to estimate the probability of transmission given contact directly from digital data of contact, recorded in a short-term Acute Care for the Elderly Unit during a 10 days period at the peak of the 2011–2012 influenza season. Various scenarios were tested. We analysed the best scenarios to predict the incidence number of influenza cases over the study period using a stochastic approach and a Susceptible-Exposed-Infectious-Removed (SEIR) model.

## Results

### Study population

This analysis relies on contact data collected by our group in a short-stay geriatric unit (20 beds)^18^ of a university hospital counting approximately 1,000 beds, located in Lyon, France. The study period was from Tuesday February 28, 2012 to Friday, March 9, 2012, during the peak of seasonal influenza in France^19^. The included data were recorded during the 10 days spanning from the 28th February 2012, at 1:00 a.m., to the 9th March 2012, at 1.00 a.m. Individuals were differentiated into 3 groups according to their role in the unit: patients (PAT), nurses (NUR) (nurses, nurses’ aides, a social counsellor and paramedical staff) and medical doctors (MD) (physicians and residents). They all received personal electronic badges to wear with an anonymous identification number.

The study protocol is detailed elsewhere^19^. Briefly, nasopharyngeal swabs were systematically collected for influenza detection by polymerase chain reaction (PCR) at the beginning and the end of the study participation time for each individual. In the case of an individual presenting one or more symptoms usually associated with influenza-like infection (ILI), additional swabs were taken for patients and HCP at onset^19^. Age, gender, influenza vaccination status, presence of ILI were recorded. Each individual was identified by his or her badge number. The infectious periods, defined in Voirin et al.^19^ for each of the individuals having confirmed infections were taken into account during the 10-day study period.

Table 1 shows the characteristics of the 3 observed groups. A total of 82 individuals took part in the study: 35 PAT and 47 HCP (32 NUR and 15 MD). All patients were 77 years old or older and 70% female. Their length of stay was 8.4 days on average with a median of 7 days. A total of 15 participants (10 PAT and 5 HCP) had confirmed influenza A (H3N2). Six individuals (3 PAT, 1 NUR and 2 MD) were prevalent contagious cases at the beginning of the study period. Four individuals contracted confirmed influenza during their stay or work schedule (3 PAT and 1 NUR). Nurses exhibited a vaccine coverage of 30%, MD 50% and PAT 55%.

**Table 1:** Characteristics of participants according to their category: PAT (patients), NUR (nurses and paramedical staff) and MD (medical doctors: physicians and residents).

	PAT	NUR	MD
n (%)	35 (43)	32 (39)	15 (18)
Age (year)			
(mean ± SD)	89.1 ± 5.9	33.9 ± 11.1	31.2 ± 14.7
Range	(76–99)	(21–61)	(21–61)
Gender			
Female	24 (69)	25 (78)	10 (67)
ILI	15 (43)	6 (19)	3 (20)
Confirmed influenza			
	10 (29)	3 (9.4)	2 (13)
Prevalent case*	3	1	2
Incident case**	3	1	0

ILI: influenza-like infection; * contagious cases at the beginning of the 10-days study period; ** acquired confirmed influenza after admission to the unit during the study period; 2 missing data for age (1 nurse, 1 medical doctor).

### Contact data

**Figure 2:**
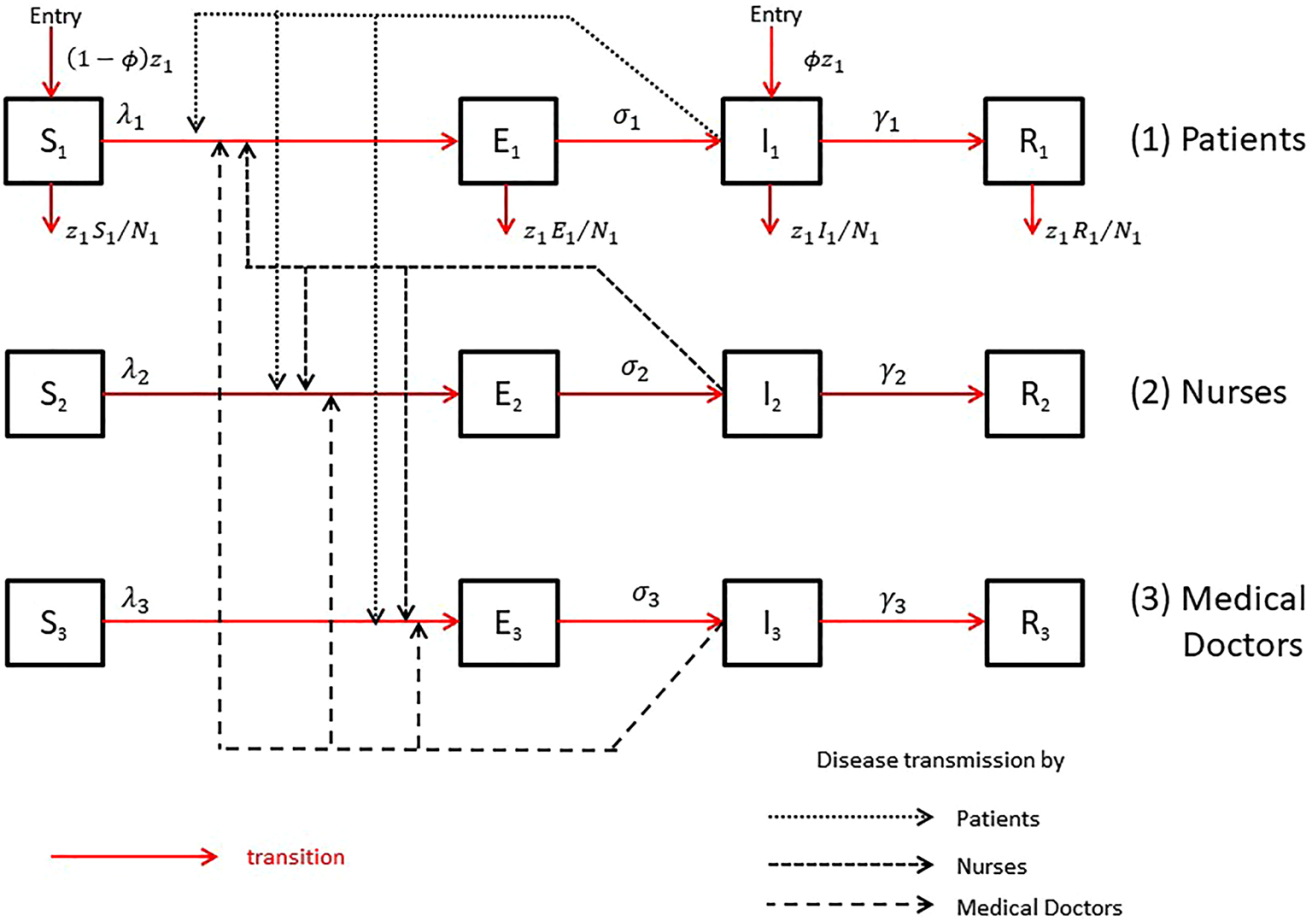
Schematic links of disease transmission and diagram of all possible transitions between the states of a SEIR model for the 3 populations: patients (1), nurses (2) and medical doctors (3). Each square corresponds to a specific state: susceptible (S), exposed (E) i.e. infected but not yet contagious, (I) infectious i.e. contagious and Removed (R) i.e. immune. Solid arrows indicate transition between the states and dotted arrows the influence of contagious individuals on the rate of transmission. The subscripts "1", "2", and "3" denote the patient, nurse and medical doctor populations, respectively. The infection rate for population $i$ is denoted by ${\lambda }_{i}$, the number of new patients entering into K2 unit (or leaving K2 unit) per day by ${z}_{1}$, the probability for patients of being in a contagious state on admission at time $t$ by $\phi$, the average duration of latent period in population $i$ by ${\sigma }_{i}^{-1}$, and the average infectious period in population $i$ by ${\gamma }_{i}^{-1}$. Patient population is stable and patients leaving K2 unit each day had same chance to be in any state.

Network contact data were collected using Radio Frequency IDentification (RFID) technology referenced previously^13,20^. Badges were worn individually. The system was tuned so that pairwise face-to-face close encounters (less than 1 m) were recorded only when they lasted longer than 20 s (s). This setup was consistent with the real case scenario leading to respiratory virus transmission. However, in this setting, two people talking next to each other might not end up in a recorded contact. The duration of the contact might be underestimated as 51% of the contacts lasted only 20 s. The distribution of their duration is reported in Fig. 2 and Table 1 of the Supplementary Information page 5. This duration would be multiplied by a factor $\left({f}_{\beta }\right)$ whose values would minimise the prediction quality of our model as explained in supplementary information pages 2 and 3. The starting and ending times of the events were recorded for each pair of established contacts. A total of 17,947 contacts were recorded during the study period with a recorded cumulative duration of 875,300 s (i.e. 243 h or 10.1 days). More detailed contact data were analysed in previous publications^19,21^.

### Transitions between states using a SEIR model

Influenza transmission in the ward is illustrated by a graphical representation of the possible transitions between different states (Fig. 2). An individual can be in 4 exclusive states: (1) *Susceptible* (at risk of contracting the disease), (2) *Exposed* to the virus (i.e. contaminated but not yet contagious), (3) *Infectious* (i.e. contagious) and (4) *Removed* (no longer able to become infected i.e. immune). Each state is represented by a compartment defining the so-called SEIR *(Susceptible-Exposed-Infectious-Removed*) model for each population (PAT/NUR/MD) and individuals could progress successively from one state to the next.

Any susceptible individual $i$ of any category (PAT, NUR, MD) can be infected by any contagious individual $j$ of any category (PAT, NUR, MD) at a specific transmission rate ${\beta }_{ij}$ per day. The number of newly infected individuals depends on these beta rates. The computation of each element ${\beta }_{ij}$ of the $\beta$ matrix is detailed in the "Method" section. We assumed no-change in the staff team during the short study period. In this first approach, we tried to keep the model as simple as possible. Then, we assumed that the random mixing inside each population and between two different ones was homogeneous, while possibly differing in vaccination status or exposition to precaution measures.

### Model parameters

Table 2 summarizes the model parameters. We estimated some parameters from the 10-day observed data and others from contact data. The average daily number of patients in the ward was estimated at 13. A mean of 2 new patients $\left({z}_{1}\right)$ entered the ward during the study and about 20% of them were already contagious. We considered that only 1/3 $\left(\tau \right)$ of the susceptible HCP were on duty at the same time and then susceptible to become infected at the same time (data taken from HCP timetable, not shown here).

**Table 2:** Definition of model parameters with their estimated value or their varying range.

Parameter	Meaning	Value	References
${N}_{1}^{tot}$	Total number of patients involved in the study	${N}_{1}^{tot}=35$	Observed
${N}_{1}$	Daily average number of patients in the ward (on any day)	${N}_{1}=$ 13	Estimated
${N}_{2}$	Number of nurses assigned to the ward (on any day)	${N}_{2}=32$	Observed
${N}_{3}$	Number of medical doctors assigned to the ward (on any day)	${N}_{3}=15$	Observed
${\overline{c} }_{ij}$	Daily average cumulative number of contacts between each individual in population $i$ and all individuals in population $j$	Values in Fig. 3	Estimated
${T}_{ij}^{\#}$	Average duration (s) per "contagious" contact between a susceptible individual in population $i$ and a contagious one in population $j$	Values in Fig. 3	Estimated
${\upsilon }_{tr}$	Average number of transmissions per hour of "contagious" contact	${\upsilon }_{T}=0.095 {h}^{-1}$	Estimated
${P}_{ij}$	Probability of transmission in time interval ${T}_{ij}$ between a susceptible individual in population $i$ and a contagious one in population $j$	Derived from ${T}_{ij}^{\#}$ and ${\upsilon }_{tr}$ (Fig. 3)	Estimated
${\beta }_{ij}$	*Per capita* transmission rate for daily "contagious" contacts between one $i$ susceptible and one $j$ contagious according to susceptibilities $\left(\alpha \right)$ and infectivities $\left(\xi \right)$ Multiplicative factor $\left({f}_{\beta }\right)$ of the transmission rate matrix	Derived from : ${P}_{ij}$ ${\alpha }_{1}=\left\{\mathrm{2,4},6\right\} ;{\alpha }_{2}=\left\{.\mathrm{5,1}\right\};{\alpha }_{3}=\left\{.25,.5\right\}; {\xi }_{11}=\{\mathrm{1,2},3\}$ ${\xi }_{12}=\left\{\mathrm{1,2}\right\}; {\xi }_{22}=\left\{.\mathrm{5,1},2\right\};{{\xi }_{13}=\left\{\mathrm{1,2}\right\}; \xi }_{33}=\{.25,.\mathrm{5,1}\};$ ${f}_{\beta }=\{\mathrm{3,4},5\}$	Estimated and Literature^22,40,41^
${\sigma }^{-1}$	Mean latent period in day (infected but not yet infectious individuals)	0.5 day	Literature^24^
${\gamma }^{-1}$	Mean infectious period for patients in days (contagious individuals)	From 1 to 4 days in step 1	Literature^34^
${z}_{1}$	Number of new patients entering the ward daily	Mean of 2 patients per day $\approx 22/10$	Estimated
$\phi$	Probability for a new patient to be in contagious state on admission	0.20 corresponding to $\approx 4/22$	Estimated
$\tau$	Proportion of HCP present in the ward at the same time	1/3	Estimated

Indices $i$ and $j$ denote the population: 1 for patients, 2 for nurses and 3 for medical doctors. Unit of time for model rates: per day, HCP: Health Care Professional (nurses and medical doctors).

**Figure 3:**
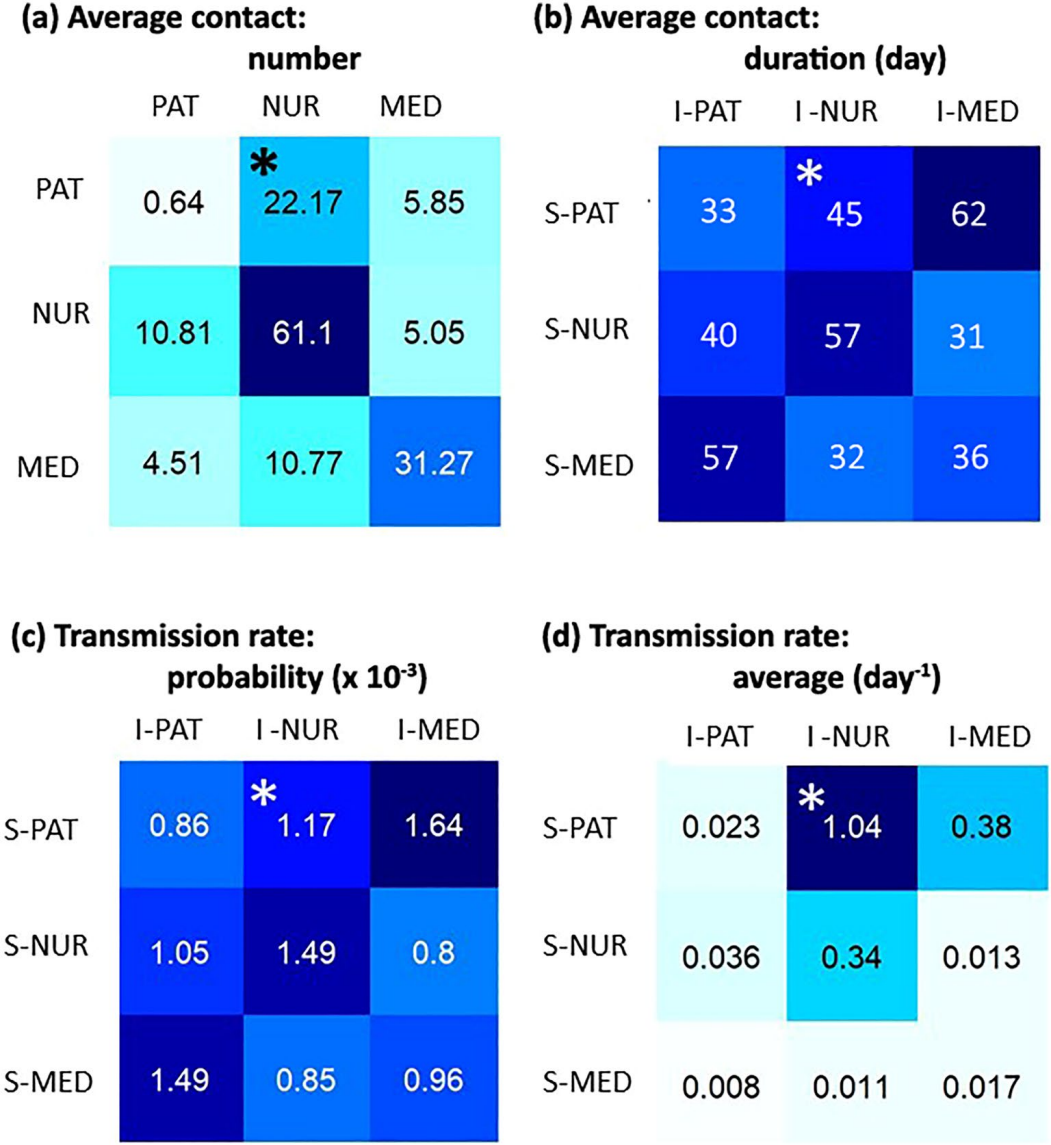
Matrices estimated from contact data and matrix of the transmission rates. **(a)** Daily average cumulative number $\left({\overline{c} }_{ij}\right)$ of contacts for an $i$-individual in line with group (Gp) $j$ in column whatever his or her state (susceptible, exposed, infectious/contagious or removed); **(b)** Average duration $\left({T}_{ij}^{\#}\right)$ per "contagious" contact during the 10-day study period, i.e. between an $i$-susceptible (S-) individual and an $j$-infectious/contagious (I-) individual, expressed in second; **(c)** Transmission probability $\left({P}_{ij}\right)$ matrix in case of "contagious" contact between two individuals (must be multiplied by 10^–3^) and **(d)** Average transmission rate per day for an $i$-susceptible (S-) individual in case of contact with any $j$-infectious (I-) individual over the 84 scenarios with a relative error less than 5% on the incidence cases number. Example of interpretation: (a) *A hospitalized patient had on average 22.17 contacts with nurses per day, (b) *A susceptible patient and a contagious nurse had contacts of 45 s on average, (c) *A susceptible patient had 1.17 × 10^–3^ chances of becoming infected in case of contact with a contagious nurse, (d) *A susceptible patient may be infected by any contagious nurse at a 1.04 rate i.e. every 23 h of "contagious" contact.

### Parameters derived from the contact data

Their estimation is described in the "Method" section. The $\beta$ matrix of transmission rates depends on the number and duration of contacts between individuals and on the probability of transmission given contact.

#### Average contact number ${\overline{{{c}}} }_{{{i}}{{j}}}$ (Fig. 3a)

The matrix of the daily average cumulative numbers of contact ${\overline{c} }_{ij}$ between an individual $i$ from one of the 3 groups and all members of a group $j$ is reported in Fig. 3a. For instance, based on this matrix, a hospitalized patient had an average of 22.17 contacts per day with nurses, whereas a nurse had an average of 10.81 contacts per day with patients. The amount of contact for an individual with a given group is specific to the category of both the individual and the group, as shown by an asymmetric matrix. The most frequent daily contacts (61.1 contacts) were within-nurses and the least frequent contacts (0.64 contact) were within-patients.

#### Average contact duration $\left({{{T}}}_{{{i}}{{j}}}^{\#}\right)$ (Fig. 3b)

The matrix of the average duration per contact $\left({T}_{ij}^{\#}\right)$ between a susceptible individual in population $i$ and a contagious individual in population $j$ is reported in Fig. 3b. The maximum average duration of these contacts is found to be between contagious MD and susceptible PAT.

#### The probability of transmission given contact $\left({\mathrm{P}}_{\mathrm{ij}}\right)$ (Fig. 3c)

This probability of transmission $\left({P}_{ij}\right)$ during a contact between an $i$-susceptible individual and an $j$-contagious individual was the highest ($1.64 .{10}^{-3}$) when a contagious MD met a susceptible PAT (Fig. 3c). These contacts are among the less frequent and the longest. HCP are more likely to transmit infection to PAT than getting one from PAT ($1.64 .{10}^{-3}$ vs $1.49 .{10}^{-3}$ for MD; $1.17 .{10}^{-3}$ vs $1.49 .{10}^{-3}$ for NUR). Nurses are more likely infected by their peers $\left(1.49 .{10}^{-3}\right)$, whereas patients do not tend to infect each other $\left(0.86 .{10}^{-3}\right)$.

#### Choice of parameters

*Susceptibility and infectivity.* The $\beta$ matrix depends on the susceptibility and the infectivity of individuals according to their category. Since no medical doctors became infected during the study period, we considered their susceptibility $\left({\alpha }_{3}\right)$ 2 to 4 times lower than the 1 default value $\left({\alpha }_{3}=\left\{0.\mathrm{25,0.5}\right\}\right)$. We considered 0.5 besides the 1 default value for nurse susceptibility $\left({\alpha }_{2}=\left\{\mathrm{0.5,1.0}\right\}\right)$. Patient susceptibility $\left({\alpha }_{1}\right)$ could be 6 times higher than the 1 default value as explained in "Method" section "Discussion about Patient susceptibility" $\left({\alpha }_{1}=\left\{\mathrm{2.0,4.0,6.0}\right\}\right)$. The ILI symptom attack rate in a patient who was in contact with contagious patients has been shown to be three times higher than the attack rate in a patient who was in contact with contagious HCP^22^ (supplementary information page 4). We considered the infectivity $\left({\upvarepsilon }_{11}\right)$ of contagious patients towards susceptible patients to be between 1 and 3 in step 1 $\left({\xi }_{11}=\left\{\mathrm{1.0,2.0,3.0}\right\}\right)$. During an epidemic, precaution measures are time-consuming for HCP^23^. An excessive workload, especially with elderly patients, might modify transmissibility if HCP are becoming less adherent to precaution measures. Besides the 1 default value, we fixed at 2 the infectivity $\left({\xi }_{12}\right)$ of contagious nurses towards susceptible patients and the infectivity $\left({\xi }_{13}\right)$ of medical doctors towards susceptible patients (i.e. ${\xi }_{12}=\left\{\mathrm{1.0,2.0}\right\}, {\xi }_{13}=\left\{\mathrm{1.0,2.0}\right\}$)^23^. We tested two opposite behaviours nurses might display while gathering together during pauses: a protective behaviour $\left({\varepsilon }_{22}=0.5\right)$ or a careless $\left({\varepsilon }_{22}=2\right)$ behaviour $\left({\varepsilon }_{22}=\left\{\mathrm{0.5,1.0,2.0}\right\}\right)$. We only considered protective behaviours between medical doctors besides 1 value $\left({\varepsilon }_{33}=\left\{0.25, 0.5, 1\right\}\right)$.

We fixed the latent period to 0.5 days to limit the number of possible model parameter combinations (Table 2). Carrat et al.^24^ reported a strong increase in viral shedding between 0.5 and 1 day and a consistent peak at 2 days after the experimental influenza infection of healthy volunteers. We allowed infectious periods to vary from 1 to 4 days in step 1 so that the generation interval of influenza remains inside the different 95% credible intervals which were estimated by Beest et al. in various settings^25^. We assumed here that infectious period for patients varied independently of HCP infectious period.

We conducted a factorial design by testing all combinations of the possible parameter values with a multiplicative coefficient set at 3, 4 and 5 in turn. This led to 62,208 different scenarios.

Because of the small population in this hospital setting (13 patients per day, 35 nurses and 15 medical doctors with only one-third of HCP on duty at the same time), events chance can have a substantial effect. As such, a stochastic approach is required to include the role of chance in the transmission process. Therefore, we chose an event-driven approach to simulate the evolution of an influenza epidemic during the study period. We relied on the Gillespie algorithm for investigating the parameter combinations using the R package GillespieSSA (function ssa) developed by Mario Pineda-Krch^26^. In this context, the probabilities of transitioning between states change over time. They depend on the current state of the populations and on the transition rate between states per day. The different transitions (Fig. 2 red lines) and their rates are reported in Table 3. These transition rates were deduced from the underlying differential equations that are reported in Supplementary Information page 7. Gillespie algorithm updates the probabilities of transitioning between states and allows simulating the evolution of the 3 populations. Successive simulations with similar parameters give different results due to the randomness. Then, we replicated them 2,000 times to report the mean of new infectious individuals appeared during the 10 days. We performed a total of 2,000 times 62,208 simulations (around 125 million).

**Table 3:** Description of model transitions and their rates.

#	Transition	Meaning	Transition rate
1	${S}_{PAT}\to {S}_{PAT}+1$	Entry of a non-contagious patient	$\left(1-\phi \right){z}_{1}$
2	${I}_{PAT}\to {I}_{PAT}+1$	Entry of a contagious patient	$\phi {z}_{1}$
3	$\left({S}_{PAT},{E}_{PAT}\right)\to \left({S}_{PAT}-1,{E}_{PAT}+1\right)$	Infection of a patient	$\left({\beta }_{11}\frac{{I}_{1}}{{N}_{1}}+{\beta }_{12}\frac{{I}_{2}}{{N}_{2}}+{\beta }_{13}\frac{{I}_{3}}{{N}_{3}}\right){S}_{1}$
4	${S}_{PAT}\to {S}_{PAT}-1$	Discharge of a susceptible patient	
5	$\left({S}_{NUR},{E}_{NUR}\right)\to \left({S}_{NUR}-1,{E}_{NUR}+1\right)$	Infection of a nurse	$\left({\beta }_{21}\frac{{I}_{1}}{{N}_{1}}+{\beta }_{22}\frac{{I}_{2}}{{N}_{2}}+{\beta }_{23}\frac{{I}_{3}}{{N}_{3}}\right)\tau {S}_{2}$
6	$\left({S}_{MED},{E}_{MED}\right)\to \left({S}_{MED}-1,{E}_{MED}+1\right)$	Infection of a medical doctor	$\left({\beta }_{31}\frac{{I}_{1}}{{N}_{1}}+{\beta }_{32}\frac{{I}_{2}}{{N}_{2}}+{\beta }_{33}\frac{{I}_{3}}{{N}_{3}}\right){\tau S}_{3}$
7	$\left({E}_{PAT},{I}_{PAT}\right)\to \left({E}_{PAT}-1,{I}_{PAT}+1\right)$	Infectivity of a patient	${\sigma }_{1}{E}_{1}$
8	${E}_{PAT}\to {E}_{PAT}-1$	Discharge of an infected but non contagious patient	${z}_{1}\frac{{E}_{1}}{{N}_{1}}$
9	$\left({E}_{NUR},{I}_{NUR}\right)\to \left({E}_{NUR}-1,{I}_{NUR}+1\right)$	Infectivity of a nurse	${\sigma }_{2}{E}_{2}$
10	$\left({E}_{MED},{I}_{MED}\right)\to \left({E}_{MED}-1,{I}_{MED}+1\right)$	Infectivity of a medical doctor	${\sigma }_{3}{E}_{3}$
11	$\left({I}_{PAT},{R}_{PAT}\right)\to \left({I}_{PAT}-1,{R}_{PAT}+1\right)$	Recovery of a patient	${\gamma }_{1}{E}_{1}$
12	${I}_{PAT}\to {I}_{PAT}-1$	Discharge of a contagious patient	${z}_{1}\frac{{I}_{1}}{{N}_{1}}$
13	$\left({I}_{NUR},{R}_{NUR}\right)\to \left({I}_{NUR}-1,{R}_{NUR}+1\right)$	Recovery of a nurse	${\gamma }_{2}{E}_{2}$
14	$\left({I}_{MED},{R}_{MED}\right)\to \left({I}_{MED}-1,{R}_{MED}+1\right)$	Recovery of a medical doctor	${\gamma }_{3}{E}_{3}$
15	${R}_{PAT}\to {R}_{PAT}-1$	Discharge of an immune patient	${z}_{1}\frac{{R}_{1}}{{N}_{1}}$

Susceptible $\left(S\right)$; Exposed (i.e. infected non contagious) $\left(E\right)$; Infectious (i.e. contagious) $\left(I\right)$; Recovered (i.e. immune) $\left(R\right).$ Indices 1 (Patient), 2 (Nurse) and 3 (Medical Doctor).

### Quality of the prediction

For each simulation, we counted the number of incident cases per category (PAT, NUR, MD). We retained the mean number of incident cases over the 2,000 simulations for each scenario as the predicted value. The quality of the model prediction for a given scenario was appreciated by comparing the observed incident cases number for each individual category^19^ with the simulated ones. The relative error was computed for each scenario ("Method" section for details). The minimum relative error was 28.8%, 3.6% and 1.9% for patient susceptibility of 2, 4 and 6, respectively. We retained the 84 scenarios that gave a relative error of less than 5%. Only 8 best scenarios had patient susceptibility of 4.

### Average transmission rate $\left({{{\upbeta}}}_{{i}{j}}\right)$ (Fig. 3d)

The $\beta$ matrix of the mean transmission rates matrix over these 84 scenarios is reported in Fig. 3d. Nurses appear to transmit infection to patients more often than medical doctors with a daily transmission rate of 1.04 days versus 0.38 for medical doctors. This means that a transmission would occur in average every 23 h during contact between contagious nurses and susceptible patients. This 1.04 rate was about three times higher than the transmissibility among nurses (rate of 0.34) and the transmissibility from contagious medical doctors towards susceptible patients. The mean reproduction number from an infectious nurse towards susceptible patients $\left({R}_{12}\right)$ was the largest among the 9 possible reproduction numbers (Supplementary Fig. 3).

## Discussion

The earliest publication, that modelled influenza in a hospital setting, estimated the parameters of contact from observed contact behaviour and the probability of transmission given contact using the modelling of an influenza epidemic in the community^7^. In this pilot study, the model we developed in the specific context of a short-stay geriatric unit allowed us to derive this probability of transmission from documented contacts between non-infected and infected individuals. Our results suggest that elderly patients have more frequent contacts, but of shorter duration, with nurses compared to medical doctors. Nurses also meet each other more often and for longer duration than medical doctors between themselves. The comparison with other reported studies may be considered carefully. Indeed, these patterns may vary according to the clinical specialty of the ward^10^.

Our results suggest that, although medical doctors were implicated in the transmission, nurses appeared to be more involved in the transmission of influenza to patients during the epidemic. The nurses encompassed nurses’ aides, a social counsellor and paramedical staff. That finding must be balanced by their essential role towards elderly patients with a necessity of close contacts in care and for humanized reasons. Nurses were less vaccinated against influenza than medical doctors (30% vs 50%). Bianchi et al. reported a 2017–2018 influenza vaccine coverage of 7% and 26% in nurses and in medical doctors, respectively, in Italy^27^. Influenza vaccine coverage was lower for nurses (42.9%), nurses assistants (26.7%) and paramedical staff (34.0%) than for medical doctors (75.5%) in nursing homes in France during the 2018–2019 influenza season^28^. This confirms that medical doctors tend to be more sensitive to vaccination campaigns over time than other health staff. The link between vaccination of HCP and the risk of nosocomial influenza in patients was reported in observational studies in France with a protective effect for patients according to the rate of vaccine coverage in HCP^27,29^. Thus, vaccination of HCP reinforced with infection control measures appeared to be a crucial key for protecting patients^29^. Our results may provide additional arguments for encouraging nurses to be vaccinated for themselves and for patients.

Some limitations should be addressed. Our model was not stratified by age as the populations were quite homogeneous. Patients were old (age > 75, mean ± SD, 90 ± $6$ years) and the mean for all HCP was 33 years (SD = 12). The potential age-based transmission effects for influenza might be tied up in the free infectivity parameter of each group.

This model disregarded vaccination status, asymptomatic patients and documented potential isolation procedure for infected patients. We had no exhaustive data available for HCP vaccination status with 23% of missing information. The vaccine effectiveness might have not been optimal during the 2011–2012 influenza epidemic in France due to a large proportion of circulating antigenic variants that differed from the vaccine strains^30^. In addition, the vaccine effectiveness differed by individual characteristics (i.e. among elderlies with underlying diseases compared to HCP in good health). Then adjustment on the vaccine composition and the individual health status as well would add complexities for transmission modelling. However, how to mitigate the viral spread in hospitals taking vaccine exposure in populations into account is an excellent question to explore further.

We may have missed some asymptomatic infected individuals because only the individuals (HCP, PAT) with at least one ILI symptom during the study participation were laboratory diagnosed. However, at the initial and distal PCR testing performed among all individuals, no asymptomatic came out. The mean length of follow-up of the patients in the ward during the 10 days study was 3.8 days with a median of 3 days, and only one out of the 3-nosocomial incident infected patients appeared asymptomatic.

This being mentioned, we believe that we did miss very few asymptomatic patients. Besides, in a systematic review including 11 studies of outbreak investigations, the asymptomatic fraction of influenza virus infections ranged from 4 to 28% with a pooled mean of 16%^31^.

Furthermore, a recent study reported a cumulative incidence of asymptomatic HCP of 47% in HCP with laboratory confirmed influenza during the 2016–2017 influenza season^32^. If we missed 50% of the asymptomatic nosocomial HCP, we should have 2 nosocomial infected HCP instead of 1 with a low impact. In addition, a community-based study from 2008 to 2014 reports that the mean levels of influenza viral RNA shedding in asymptomatic cases was lower than in symptomatic cases, its duration was shorter and declined more rapidly as well^33^. These findings suggest that asymptomatic individuals had lower potential for transmitting the virus than symptomatic ones. We did not think that adding one infected HCP would change very much our results, and thus we did not take them into account.

A more sophisticated model incorporating various ranges of percentages of asymptomatic cases^24,34^ should be considered in a future work. Indeed, the infectivity of a given subject varies with time and is proportional to the viral load^35^. Here, to keep the model simple, we did not take into account these variations and only conserved one compartment for contagious subjects.

The isolation protocols for patients, the precaution measures (hand hygiene, mask) and their respect were not collected during the study. However, the surveillance of nosocomial influenza is ongoing in this unit since 2004^22^. Then, HCP are well trained and very aware on this risk. Consequently, taking isolation protocols into account would not have changed our conclusions substantially.

A fourth population involved in the pattern of transmission would be the visitors. Because of the complexity of collecting valid information on contacts for this population (no badges), they were not included in the model of this pilot analysis.

These limitations had less impact on the results with our model than with the classical ones for which the transmission of infection given contact is fixed. Here, the transmission of infection was computed from several free parameters to take the various populations and their contact patterns into consideration. A future study should thus be planned with serological confirmation influenza completed by systematic collection of vaccination data status and the precise timetable of HCP. More data and more sophisticated models should be collected and performed to validate these results. In addition, nosocomial viral respiratory infection caused by influenza or more recently by SARS-COV-2 will have major consequences including morbidity, access and organisation of care, length of stay and financial costs^36–39^. The transmission model results may be considered as strong contributions for proposing adapted preventive measures aiming to control viral spread in hospitals.

## Method

In this section, we explained how the viral data were collected. We detailed the steps that allowed us estimating the matrix of the per capita transmission rates. We discussed the patient susceptibility according to the estimation of the number of transmissions per hour of "contagious" contacts.

### Virological data

Nasopharyngeal swabs were taken to confirm influenza infection. These swabs were sent to the Southern French National Reference Laboratory for Influenza (Lyon, France) and were analysed for influenza A and B viruses by real-time polymerase chain reaction (PCR; Respiratory Multi-Well system R-gene®, Argene, Verniolle, France)^19^.

### Matrix of the per capita transmission rate $\left({{\varvec{\upbeta}}}_{\mathbf{i}\mathbf{j}}\right)$

The coefficient ${\beta }_{ij}$ is the probability that a contagious individual of population $j$ infects a susceptible individual of population $i$ per day, and is defined as follows^11^.

$${\beta }_{ij}={\overline{c} }_{ij}\times {\alpha }_{i}\times {\xi }_{ij}\times {P}_{ij} , (1)$$

with ${\overline{c} }_{ij}$ denoting the average number of contacts per time unit for an individual in population $i$ with all individuals in population $j$; ${\alpha }_{i}$ the susceptibility of individuals in population $i$; ${\xi }_{ij}$ the infectivity of contagious individuals in population $j$ towards susceptible individuals in population $i$, and ${P}_{ij}$ the probability of transmission per contact between a susceptible individual in population $i$ and a contagious individual in population $j$^11^

The default values for susceptibility and infectivity were 1. Patient $\left({\alpha }_{1}\right)$, nurse $\left({\alpha }_{2}\right)$ and medical doctor $\left({\alpha }_{3}\right)$ susceptibilities, patient-patient infectivity $\left({\xi }_{11}\right)$ and HCP infectivity $\left({\xi }_{12},{\xi }_{22},{\xi }_{13}, {\xi }_{33}\right)$ towards patients or HCP were allowed to vary during the calibration process. The computation of ${\overline{c} }_{ij}$ and ${P}_{ij}$ are detailed below.

### The average number ${\overline{\mathbf{c}} }_{\mathbf{i}\mathbf{j}}$

The daily average cumulative number of contacts for a patient with population $j$ is given by the following formula:

$${\overline{c} }_{1j}=\frac{\sum_{k=1}^{{Np}_{1}}\left(\frac{\sum_{t=1}^{{L}_{k}}\sum_{m=1}^{{Np}_{j}}{c}_{kmt}}{{L}_{k}}\right)}{{Np}_{1}},$$

with ${Np}_{1}$ denoting the number of patients (n = 35) included in the study and ${Np}_{2}$ or ${Np}_{3}$ the number of nurses (n = 32) or medical doctors (n = 15) on duty in the ward during the study period; ${L}_{k}$ the length of stay in days of the k-th patient and ${c}_{kmt}$ the number of contacts between the k-th patient and the m-th individual in population $j$ on day $t$.

In the case of nurses and medical doctors, the formula was:

$${\overline{c} }_{ij}=\frac{\sum_{k=1}^{{Np}_{i}}\sum_{m=1}^{{Np}_{j}}{c}_{km}}{{Np}_{i}\times {T}_{study}},$$

with ${Np}_{i}={N}_{i}$ and ${c}_{km}$ denoting the number of contacts between the k-th individual in the case of nurses and medical doctors $i$ and the m-th individuals in population $j$ during the whole study period.

### Estimation of the probability ${\mathbf{P}}_{\mathbf{i}\mathbf{j}}$ of contact transmission

#### Definition

The transmission probability matrix $\left({P}_{ij}\right)$ renders the probabilities ${P}_{ij}$ that a susceptible individual in population $i$ became infected after a contact of duration ${T}_{ij}^{\#}$ with any contagious individual of population $j$. The probability ${P}_{ij}$ was considered as a function of the contact duration ${T}_{ij}^{\#}$.

$${P}_{ij}=1-{e}^{-{\upsilon }_{T}{T}_{ij}^{\#}}\approx {{\varvec{\upsilon}}}_{{\varvec{t}}{\varvec{r}}}{{\varvec{T}}}_{{\varvec{i}}{\varvec{j}}}^{\#}.$$

The probability $\left({P}_{ij}\right)$ that at least 1 transmission occurs during a contact in time interval ${T}_{ij}^{\#}$ is $\left(1-{e}^{-{\upsilon }_{T}{T}_{ij}^{\#}}\right)$ assuming the number of transmissions during a contact of ${T}_{ij}^{\#}$ duration follows a Poisson distribution of mean $\left({\upsilon }_{tr}{T}_{ij}^{\#}\right)$, with ${\upsilon }_{tr}$ being the average number of transmissions per unit of "contagious" contact duration (seconds) between a susceptible $i$ and a contagious $j.$

#### Estimation of ${{\varvec{T}}}_{{\varvec{i}}{\varvec{j}}}^{\#}$

The contact duration ${{\varvec{T}}}_{{\varvec{i}}{\varvec{j}}}^{\#}$*.* was estimated by the average duration for each contact between all susceptible individuals in population $i$ and all contagious individuals in population $j$ during their contagious period:

$${T}_{ij}^{\#}=\frac{1}{\sum_{k=1}^{{Ns}_{i}}\sum_{q=1}^{{Nf}_{j}}{n}_{kq}}\left(\sum_{k=1}^{{Ns}_{i}}\sum_{q=1}^{{Nf}_{j}}\sum_{c=1}^{{n}_{kq}}{d}_{kqc}\right),$$

with ${Ns}_{i}$ denoting the number of individuals in population $i$ who were susceptible; ${Nf}_{j}$ the number of individuals in population $j$ who were contagious during the study period $T$; ${n}_{kq}$ the number of contacts between the k-th individual in population $i$ when he or she is susceptible and the q-th individual in population $j$ during his or her infectious period, and ${d}_{kqc}$ the duration of each c-th "contagious" contact.

#### Estimation of ${{\varvec{\upsilon}}}_{{\varvec{T}}}$

The number of transmissions per hour of "contagious" contact was estimated as follows:

$${\upsilon }_{T}=\frac{{Nf}^{\#}}{{\sum }_{i=1}^{3}{\sum }_{j=1}^{3}\sum_{k=1}^{{Ns}_{i}}\sum_{q=1}^{{Nf}_{j}}\sum_{c=1}^{{n}_{kq}}{d}_{kqc}},$$

with ${Nf}^{\#}$ the number of incident cases that became contagious during the study period. The average number ${\upsilon }_{T}$ of transmissions per time unit of "contagious" contacts was supposed to be independent of the category of the population in this computation. This unrealistic hypothesis would be counteracted by the varying susceptibility $\left({\alpha }_{i}\right)$ across the three populations.

Only 4 nosocomial infections (3 patients and 1 nurse) occurred during the study period and the total cumulative "contagious" contacts over all populations were 152,200 s. The number ${\upsilon }_{tr}$ of transmissions per hour was therefore approximately **0.095** $\left(\frac{4\times \mathrm{3,600}}{\mathrm{152,200}}\right)$.

### Discussion about PATIENT susceptibility

This number ${{\varvec{\upsilon}}}_{{\varvec{t}}{\varvec{r}}}$ of transmissions per hour of "contagious" contacts of **0.095** was considerably lower than those reported in the literature for influenza. In comparison, this number ${\upsilon }_{tr}$ was taken at 0.20 transmissions per hour of contact by Del Valle et al.^11^ in case of an influenza epidemic in the community and estimated at 0.56 per hour by Salathé et al.^40^ because it approximates the time-dependent attack rate in an outbreak of influenza aboard a commercial airliner^41^. This latter ${\upsilon }_{tr}$ value is approximately 6 times higher than our own. Indeed, we considered that the self-protective behaviour against influenza transmission adopted by the passengers aboard the airliner might be similar to hospitalized patient behaviour and less careful than HCP behaviour. By default, all individual susceptibility was fixed at 1 but patient susceptibility $\left({\mathrm{\alpha }}_{1}\right)$ can reach 6 according to Salathé et al.^40^ The 2, 4 and 6 values were tested as the potential patient susceptibility in the model.

### Relative error of prediction per scenario

We assessed the prediction power of the model for a given parameter set by comparing the average number of incident cases over 2,000 simulations with the observed case number per category of individuals as follows:

$$RE\left(\%\right)=100\times \frac{\sum_{i=1}^{3}\left|{O}_{i}-\frac{{\sum }_{k=1}^{\mathrm{2,000}}{PN}_{ik}}{\mathrm{2,000}}\right|}{\sum_{i=1}^{3}{O}_{i}},$$

with $i$ denoting population $i$ (1:PAT; 2:NUR; 3:MD); ${O}_{i}$ the Observed number of incident cases in population $i$ during the 10-day study period and ${PN}_{ik}$ the average of the Predicted Number of incident cases in population $i$ for the simulation $k$ over all 2,000 simulations.

### Data analysis

Data analysis was performed using R language version 4.1.0 freely available at https://cran.r-project.org and Octave version 4.2.1 freely available at http://www.octave.org.

### Ethics and Privacy

All participants signed an informed consent form after having acknowledged the details and aims of the study as explained elsewhere^19^. All experimental protocols were approved by the French national bodies responsible for ethics and privacy: the National Commission for Information Technology and Civil Liberties (http://www.cnil.fr) and the Committee for the Protection of Individuals (http://www.cppsudest2.fr) of the hospital of Lyon. All methods were performed in accordance with the relevant guidelines and regulations.

## Supplementary Information

Supplementary Information.

## Data Availability

The data and the source code that support the findings of this study are available from the corresponding author upon reasonable request.

## Abbreviations

ILI: Influenza-like illness
HCP: Health care professional
SEIR: Susceptible-exposed-infectious-removed
PAT: Patients
NUR: Nurses
MD: Medical doctors
