# Supplementary material for: Influenza transmissibility among patients and health-care professionals in a geriatric short-stay unit using individual contact data
Source: Sci Rep. 2023 Jun 29;13:10547. doi: 10.1038/s41598-023-36908-5 (PMC10310843; doi:10.1038/s41598-023-36908-5)
Supplement: Supplementary file 1 — Supplementary Information. [file 41598_2023_36908_MOESM1_ESM.pdf]

Supplementary Information for

Influenza transmissibility among patients and health-care professionals in a geriatric short-stay unit using individual contact data

Marie-Paule Gustin*, Laurent Pujo-Menjouet, and Philippe Vanhems.

*Corresponding author. Email: marie-paule.gustin@univ-lyon1.fr

Supplementary information:

Page

- Determination of the β matrix multiplicative factor (f_β) 2
- Evaluation of the susceptibility of patients faced to contagious patients 4
- Duration of the contacts between two individuals 5
- Estimation of the next generation matrix at the beginning of the study...............6
- Underlying differential equations system 7
- References 8

Determination of the $\boldsymbol{\beta}$ matrix multiplicative factor $(\boldsymbol{f_\beta})$

The average duration of Hospital Care Professional (HCP) contacts are extremely short in our data compared with those reported in the literature. We observed indeed a mean duration of 0.77 min (46 s) ranging from 0.33 to 12 min and a median of 0.33 [IQR, 0.33-1.0] min. But, in a study dealing with HCP exposure to tuberculosis infected patients[1], the average duration of a HCP visit manually recorded in a patient room was 3 min 26s ranging from 10 s to 18 min i.e. 4.5 times higher than our results on average. In another paper of the same previous team, the median interaction duration between HCP and patients was 2.5 [IQR, 1.6-7.7] min by radiofrequency identification[2] i.e. from 6 to 7.6 times higher than our median duration. Contacts within 1 meter were recorded in our study whereas the maximal distance was from 1.5 to 2.74 m (9 feet) in other studies[1,3–6], i.e. almost 3 times farther. Furthermore, face-to-face contact means that two persons sitting next to each other who talk might not be recorded as a contact. Consequently, a ward assistant busy in a patient room for a while might not be recorded at all in our data.

To counteract these drawbacks, we computed the average relative error of prediction of our model by varying the multiplicative factor (f_β) between 1 and 10 step 1. The transmission matrix (β_{ij}) was estimated with all susceptibility and infectivity parameters set to the default value 1, the latency period was fixed at 0.5 day and the infectious period varied from 1 to 4 step 1 for patients and HCP. This led to test 80 parameter combinations (scenarios) i.e. $10 \times 2 \times 4$. For each scenario, 2,000 simulations were performed using the stochastic SEIR model. The mean of the predicted number of incident cases by subject category (patient, nurse, medical doctors) was computed over the 2,000 simulations for each scenario. The mean prediction error for a given scenario was the average over the subject category of the absolute difference between the numbers of observed and mean predicted incidence cases. The relative error (RE) of prediction per scenario was then computed as follows:

$$RE(\%) = 100 \times \frac{\sum_{i=1}^{3} \left| O_i - \frac{\sum_{k=1}^{2,000} PN_{ik}}{2,000} \right|}{\sum_{i=1}^{3} O_i},$$

with i denoting population i (1: patients; 2: nurses; 3: medical doctors); O_i the Observed number of incident cases in population i during the 10-days study period and PN_{ik} the average of the Predicted Number of incident cases in population i for the simulation k over all 2,000 simulations.

The average relative error of prediction per multiplicative factor f_β is reported in Fig. 1 below.

Supplementary Figure 1. Average relative error of prediction obtained for the different multiplicative factors (f_β).

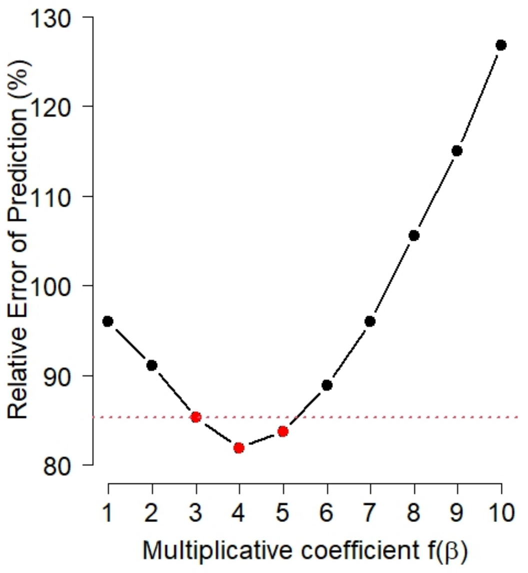

The best predictions were obtained with the model using a multiplicative factor of 3, 4 or 5.

This means that a recorded face-to-face contact of 20 s corresponds to a close presence lasting from 1 min to 1 min 40 s.

Then the values of 3, 4 and 5 corresponded to the three minimum relative errors of prediction on average. These values were thus tested in the model as multiplicative factor of the coefficients β_{ij} of the β_s matrix as reported in the Method section.

Evaluation of the susceptibility of patients faced to contagious patients

In Vanhems et al.[7] our team estimated

1) a relative risk (RR) of 5.48 for influenza-like illness (ILI) in hospitalized patients in case of exposure to at least one health care worker (HCW) and no patients with ILI in the ward compared with exposure to no patients and no HCW with ILI in the ward and
2) a RR of 17.96 for ILI in hospitalized patients in case of exposure to at least one patient and no HCW with ILI compared with exposure to no patients and no HCW with ILI in the ward.

These results are summarized in the figure included in the paper of Vanhems et al.[7]

This led to a RR of approximately 3 for ILI in hospitalized patients in case of exposure to patients with ILI in the ward compared with exposure to HCW with ILI in the ward:

$$RR = \frac{17.96}{5.48} = 3.28 \approx 3$$

Then, we considered that patient infectivity towards susceptible patients might be 3-fold the HCW infectivity toward susceptible patients. The patient infectivity toward susceptible patients denoted by ε_{11} parameter therefore varied between 1 and 3.

Duration of the contacts between two individuals

Contact duration ranged from 20 seconds to almost 1 hour 51 minutes. The longest contact duration was between two interns (residency program) who were on ward duty by night. Over the 17,947 contacts, 99.1% (17,791) of them lasted no more than 5 minutes. Supplementary Fig.2 gives the distribution of their duration.

Supplementary Figure 2. Distribution of the 17,791 contact durations equal to 5 minutes or shorter

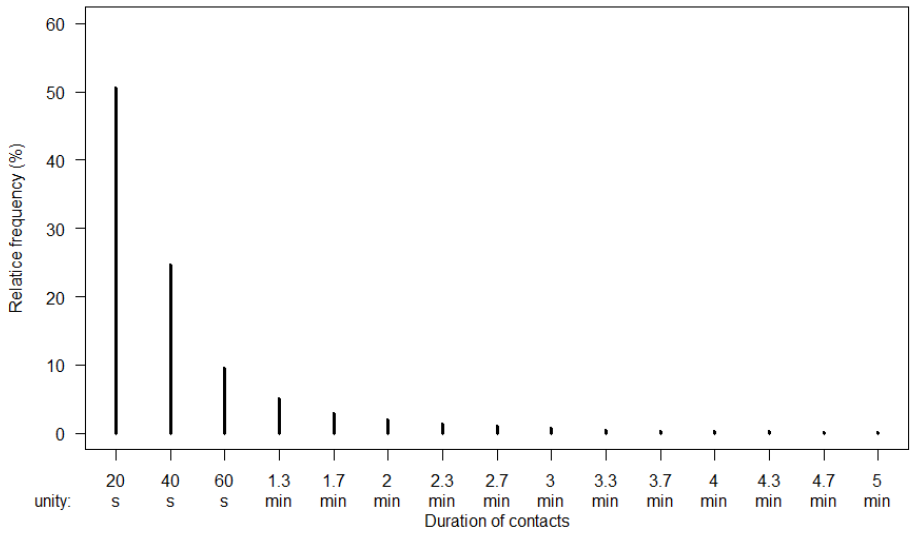

50.6 % of the contacts (9080/17,947) lasted 20 s.
75.3% of the contacts (13,506/17,947) lasted 20 s or 40 s.
94.5% of the contacts (16,963/17,947) lasted 2 min or less.
99.8% of the contacts (17,918/17,947) lasted 10 min or less.

Supplementary Table 1 below gives the distribution of the extreme contact durations.

Supplementary Table 1. Distribution of the extreme contact durations.

Duration	Frequency	Relative frequency (%)
<= 5 min	17,791	99.1
] 5, 10] min	127	0.71
] 10, 15] min	15	0.084
]15, 60] min	11	0.061
]1 , 2] h	3	0.017

Estimation of the next generation matrix at the beginning of the study

We estimated the next generation matrix according to Diekmann et al.[8,9]. We computed then the mean for each matrix element.

Nurses appeared to be involved in patient infection. The mean reproduction number from an infectious nurse toward susceptible patients (R_{12}) was the largest among the 9 possible reproduction numbers (Supplementary Fig. 3).

Supplementary Figure 3. Effective reproduction number R matrix obtained on average for the 84 best scenarios with relative error less than 5%.

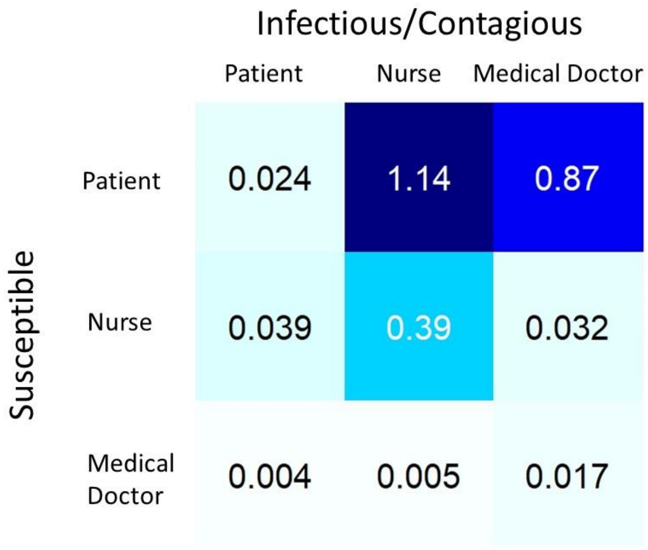

Underlying differential equations system

The transition rates (see Table 3 in the main text) were deduced from the following ordinary differential equations that describe the dynamics of the diagram of the transitions between the states given in Fig. 2 in the main text.

For patient, $i = 1$

$$\dot{S}_{1,t} = (1-\phi)z_1 - \left(\frac{z_1}{N_1} + \beta_{11}\frac{I_{1,t}}{N_1} + \beta_{12}\frac{I_{2,t}}{N_2} + \beta_{13}\frac{I_{3,t}}{N_3}\right)S_{1,t}\ ,$$

$$\dot{E}_{1,t} = \left(\beta_{11}\frac{I_{1,t}}{N_1} + \beta_{12}\frac{I_{2,t}}{N_2} + \beta_{13}\frac{I_{3,t}}{N_3}\right)S_{1,t} - \left(\frac{z_1}{N_1} + \sigma_1\right)E_{1,t}\ ,$$

$$\dot{I}_{1,t} = \phi z_1 + \sigma_1 E_{1,t} - \left(\frac{z_1}{N_1} + \gamma_1\right)I_{1,t}\ ,$$

$$\dot{R}_{1,t} = \gamma_1 I_{1,t} - \frac{z_1}{N_1}R_{1,t}\ .$$

For HCP, $i = 2$ (nurses) or 3 (medical doctors):

$$\dot{S}_{i,t} = -\left(\beta_{i1}\frac{I_{1,t}}{N_1} + \beta_{i2}\frac{I_{2,t}}{N_2} + \beta_{i3}\frac{I_{3,t}}{N_3}\right)\tau S_{i,t}\ ,$$

$$\dot{E}_{i,t} = \left(\beta_{i1}\frac{I_{1,t}}{N_1} + \beta_{i2}\frac{I_{2,t}}{N_2} + \beta_{i3}\frac{I_{3,t}}{N_3}\right)\tau S_{i,t} - \sigma_i E_{i,t}\ ,$$

$$\dot{I}_{i,t} = \sigma E_{i,t} - \gamma_i I_{i,t}\ ,$$

$$\dot{R}_{i,t} = \gamma_i I_{i,t},$$

where σ_i and γ_i denote the contagious individuals and removing rate in i population respectively; z_1 the number of daily new patients entering the ward; and ϕ the percentage of those patients already contagious. The latent period lasts ${\sigma_i}^{-1}$ unit of time (days) in average and the average contagious period ${\gamma_i}^{-1}$ days for individuals of i population. For HCP, we estimated that roughly a third of them $(\tau = 1/3)$ were on duty at the same time, using anonymous timetable corresponding to the same period study.

Any i population was supposed to remain constant:

$$S_{i,t} + E_{i,t} + I_{i,t} + R_{i,t} = N_i.$$

With N_i the number of hospitalized patients in the ward $(i = 1)$ or the total number of HCP ($i \in \{2,3\}$).

The initial conditions were taken as follows: 6 individuals (3 PAT, 1 NUR and 2 MD) were prevalent contagious cases at the beginning of the study period.

In the model, the population of patients at time t corresponds to patients hospitalized in the ward at time t and the populations of HCP (nurses and medical doctors) correspond to HCP assigned to the ward during the study period.

An average fixed number of z_1 patients could enter the ward per day into two possible states, susceptible or contagious, with probability $(1 - \phi)$ and ϕ respectively. A number of z_1 patients left the ward per day in any of the 4 possible states (Susceptible/Exposed/Infectious/Removed) with the same probability. The HCP populations were closed assuming no-change in the staff team during the short study period.

We did not take the transmission of influenza in the community into account because of the short stay of patients in the ward. Indeed, we assumed the cautiousness of the families when visiting elderly individuals.

References

1. Friggeri, A. *et al.* Reconstructing social interactions using an unreliable wireless sensor network. *Comput. Commun.* **34**, 609–618 (2011).
2. Lucet, J.-C. *et al.* Electronic Sensors for Assessing Interactions between Healthcare Workers and Patients under Airborne Precautions. *PLoS ONE* **7**, e37893 (2012).
3. Kazandjieva, M. A. *et al.* Experiences in measuring a human contact network for epidemiology research. in *Proceedings of the 6th Workshop on Hot Topics in Embedded Networked Sensors - HotEmNets '10* 1 (ACM Press, 2010). doi:10.1145/1978642.1978651.
4. Leecaster, M. *et al.* Estimates of social contact in a middle school based on self-report and wireless sensor data. *PloS One* **11**, e0153690 (2016).
5. Mastrandrea, R., Fournet, J. & Barrat, A. Contact patterns in a high school: a comparison between data collected using wearable sensors, contact diaries and friendship surveys. *PLOS ONE* **10**, e0136497 (2015).
6. Smith, D. R. M. *et al.* Optimizing COVID-19 surveillance in long-term care facilities: a modelling study. *BMC Med.* **18**, 386 (2020).
7. Vanhems, P. *et al.* Risk of influenza-like illness in an acute health care setting during community influenza epidemics in 2004-2005, 2005-2006, and 2006-2007: a prospective study. *Arch. Intern. Med.* **171**, (2011).
8. Diekmann, O., Heesterbeek, J. A. P. & Roberts, M. G. The construction of next-generation matrices for compartmental epidemic models. *J. R. Soc. Interface* **7**, 873–885 (2010).
9. Diekmann, O., Heesterbeek, J. A. P. & Metz, J. A. J. On the definition and the computation of the basic reproduction ratio R_0 in models for infectious diseases in heterogeneous populations. *J. Math. Biol.* **28**, (1990).
